# Evaluating Spatial, Cause-Specific and Seasonal Effects of Excess Mortality Associated with the COVID-19 Pandemic: The Case of Germany, 2020

**DOI:** 10.1007/s44197-023-00141-0

**Published:** 2023-08-04

**Authors:** Michael Mühlichen, Markus Sauerberg, Pavel Grigoriev

**Affiliations:** Federal Institute for Population Research (BiB), Friedrich-Ebert-Allee 4, 65185 Wiesbaden, Germany

**Keywords:** COVID-19, Excess mortality, Causes of death, Regional differences, Germany

## Abstract

**Background:**

Evaluating mortality effects of the COVID-19 pandemic using all-cause mortality data for national populations is inevitably associated with the risk of masking important subnational differentials and hampering targeted health policies. This study aims at assessing simultaneously cause-specific, spatial and seasonal mortality effects attributable to the pandemic in Germany in 2020.

**Methods:**

Our analyses rely on official cause-of-death statistics consisting of 5.65 million individual death records reported for the German population during 2015–2020. We conduct differential mortality analyses by age, sex, cause, month and district (N = 400), using decomposition and standardisation methods, comparing each strata of the mortality level observed in 2020 with its expected value, as well as spatial regression to explore the association of excess mortality with pre-pandemic indicators.

**Results:**

The spatial analyses of excess mortality reveal a very heterogenous pattern, even within federal states. The coastal areas in the north were least affected, while the south of eastern Germany experienced the highest levels. Excess mortality in the most affected districts, with standardised mortality ratios reaching up to 20%, is driven widely by older ages and deaths reported in December, particularly from COVID-19 but also from cardiovascular and mental/nervous diseases.

**Conclusions:**

Our results suggest that increased psychosocial stress influenced the outcome of excess mortality in the most affected areas during the second lockdown, thus hinting at possible adverse effects of strict policy measures. It is essential to accelerate the collection of detailed mortality data to provide policymakers earlier with relevant information in times of crisis.

**Supplementary Information:**

The online version contains supplementary material available at 10.1007/s44197-023-00141-0.

## Introduction

COVID-19-related mortality varied considerably across Europe in 2020, with Southern and Eastern Europe widely showing higher levels than the north of Europe [1–4]. Although located in the middle between hot and cold spots of excess mortality, Germany as a whole experienced only a slight decline in life expectancy at birth—compared to most other European countries-from 81.20 years in 2019 to 81.01 in 2020 for both sexes combined [5]. However, this is not true for all German regions. First spatial analyses revealed that the regions that are in close proximity to Czechia and Poland, which were hot spots of the second wave of the COVID-19 pandemic—experienced significantly higher excess mortality compared to the rest of Germany [6–9]. For instance, the federal state of Saxony in the south of eastern Germany showed comparatively high excess mortality in 2020 (-0.7 decrease in life expectancy), whereas Schleswig–Holstein in the north showed no excess mortality at all (+ 0.1 increase in life expectancy).

Although all-cause mortality analyses on national or broad regional levels are inevitably associated with the risk of masking important subnational differentials, e.g. in connection with border regions, and hampering targeted health policies, there is no study so far for Germany that focuses on a smaller regional level and considers causes of death. Even from a worldwide perspective, there are only very few studies that consider both the spatial and the cause-of-death dimension [10, 11].

This is related to the lengthy process of data collection and preparation in statistical offices. Given the urgency to analyse the consequences of the pandemic to help find adequate measures to combat it, scientists had to use preliminary and often incomplete or estimated data on all-cause death counts, which became available with comparatively short delay for many nations. Meanwhile, however, more detailed data for 2020 by cause of death, season, age, sex and region have become available in many European countries.

As we can assume that the regional differences in excess mortality in Germany to a certain degree mirror a Europe-wide gradient, Germany represents a fascinating context for such comprehensive analysis. The findings as to which age groups and which causes of death determined the regional and seasonal pattern of excess mortality might allow conclusions beyond the German context.

Therefore, the objective of this paper is to estimate excess mortality for men and women in Germany in 2020 for the first time on the NUTS-3 level (400 districts/counties) and by cause of death, season, age and sex. This allows us to identify the spatial clusters of elevated mortality and disentangle the effects of important determinants.

## Data and Methods

### Data Preparation

Our analyses are based on official data. We obtained regional population and death counts by age and sex from 1990 onwards from the German Federal and State Statistical Offices and accessed the German cause-of-death statistics, consisting of 5.65 million death records between 2015 and 2020, in Wiesbaden at the Research Data Centre of Germany’s statistical offices. To use a coherent time series, we applied the current administrative division as of 2022 with 400 spatial units to previous years and harmonised age-specific population counts prior to 2011 to eliminate the census 2011 break [12, 13]. Age is divided into 5-year groups (0, 1–4, 5–9, …, 85–89, 90 +).

Causes of death have been recorded in Germany according to the tenth revision of the International Classification of Diseases (ICD-10) since 1998. We chose ten causal groups for our study (Table 1). For the hot-spot analyses that compare 2020 with previous years, we had to include COVID-19 in a broader disease group because the disease had not broken out in Germany before 2020. Even though COVID-19 is a respiratory disease that in severe cases often involves pneumonia [14], we decided to add it to ‘other diseases’ because we wanted to analyse respiratory diseases—and pneumonia in particular—separately to explore whether there were regional coding differences related to COVID-19.

**Table 1:** Cause-of-death groups by ICD-10 codes.

Cause-of-death groups	ICD-10
All causes	A00-R99, V00-Y99
Neoplasms	C00-D48
Cardiovascular diseases (CVD)^a^	I00-I99
Respiratory diseases^a^	J00-J99
- Pneumonia	J12-J18
Digestive diseases	K00-K93
Mental and nervous disorders	F00-G99
External causes	V00-Y99
COVID-19	U07.1, U07.2
Other diseases	All other codes

^a^For the seasonal analyses, we further separated CVD into ischaemic heart disease (I20-I25), cerebrovascular disease (I60-I69) and other CVD (I00-I19, I26-I59, I70-I99), and respiratory diseases into pneumonia (J12-J18) and other respiratory diseases (J00-J11, J19-J99).

### Baseline

There has been a lot of discussion on the methodological challenges for choosing an appropriate baseline when calculating excess mortality. Previous studies have examined the impact of different mortality indicators, reference periods and statistical models on the sensitivity of excess mortality [15–20]. For our analyses, however, we declined to estimate artificial baselines based on these approaches because they get increasingly insecure on a smaller regional level, especially when causes of death are considered. Furthermore, we are more interested in the spatial variation than on the ‘correct’ level of excess mortality. Therefore, we used previous calendar years (2015–2019) as our baseline. We chose a five-year period to account for random annual fluctuations, which are high on a small spatial level.

### Methods

We first estimated life expectancy at birth to present mortality trends for Germany’s 16 federal states (‘Bundesländer’) by sex from 1990 to 2020 according to the methodology of the Human Mortality Database [21] and decomposed the differences between 2020 and 2015/2019 into the contributions of selected age groups using the stepwise-replacement algorithm [22] via the *DemoDecomp* R package [23]. To identify seasonal patterns, we calculated seasonality indices, i.e. ratios of the monthly numbers of deaths per day and the annual numbers of deaths per day, for 2015/2019 and 2020 by causal group and federal state.

To show the change between the two periods on the district (‘Kreise’) level, we calculated standardised mortality ratios (SMRs) for men and women, relating the mortality observed in 2020 to the mortality expected based on the 2015/2019 pattern. For cause-specific analyses, we computed standardised death rates (SDRs) for the two periods by cause, sex and region per 1 million people, using the European Standard Population 2013. We calculated SDR ratios by dividing the SDR for 2020 by the SDR for 2015/2019 for each region by causal group and sex. For both SMRs and SDR ratios, values higher than 1.0 indicate excess mortality as compared to the baseline period. In addition, we calculated the difference in cause-specific SDRs between the two periods, focusing on those regions that were either most or least affected by excess mortality, to show which causes were driving the changes compared to previous years.

Based on the SDR ratios, we identified spatial clusters of significantly higher and lower cause-specific mortality (hot spots and cold spots), as compared to the other regions under study, relying on the Getis-Ord Gi* statistic [24]. To be a statistically significant hot (or cold) spot, a spatial unit should not only have a high (or low) value but also be surrounded by other regions with high (or low) values. The local sum for a given district and its neighbouring districts is evaluated against the sum of all districts. If the observed local sum is highly different from the expected local sum, then this difference is not the result of random chance and the corresponding z-score is statistically significant [25].

For supplementary analyses, we conducted spatial regression models to estimate the area-level correlations of cause-specific SDRs and SDR ratios—for both sexes combined—with pre-pandemic contextual factors [8, 26]. We applied queen neighbourhood as spatial weight matrix [27] because distance-based weights are not suitable for German districts due to their large variation in size. We conducted ordinary least squares (OLS), spatial lag and spatial error models. We chose the spatial error model for our interpretation because it considers the spatial dependence in a spatially correlated error term in the regression equation and allows for the modelling of spatial autocorrelation [28]. Furthermore, it showed the best fit for most of our models, as suggested by the outcomes of Moran’s I, LaGrange multiplier tests and Akaike information criterion (AIC).

We performed all calculations in *R* and all spatial analyses in *ArcMap*, using the German administrative regional shapefile from the Federal Agency for Cartography and Geodesy as of 31 December 2021 based on the Universal Transverse Mercator projection (UTM32s) [29].

## Results

### Life Expectancy Changes by Federal State

**Fig. 1:**
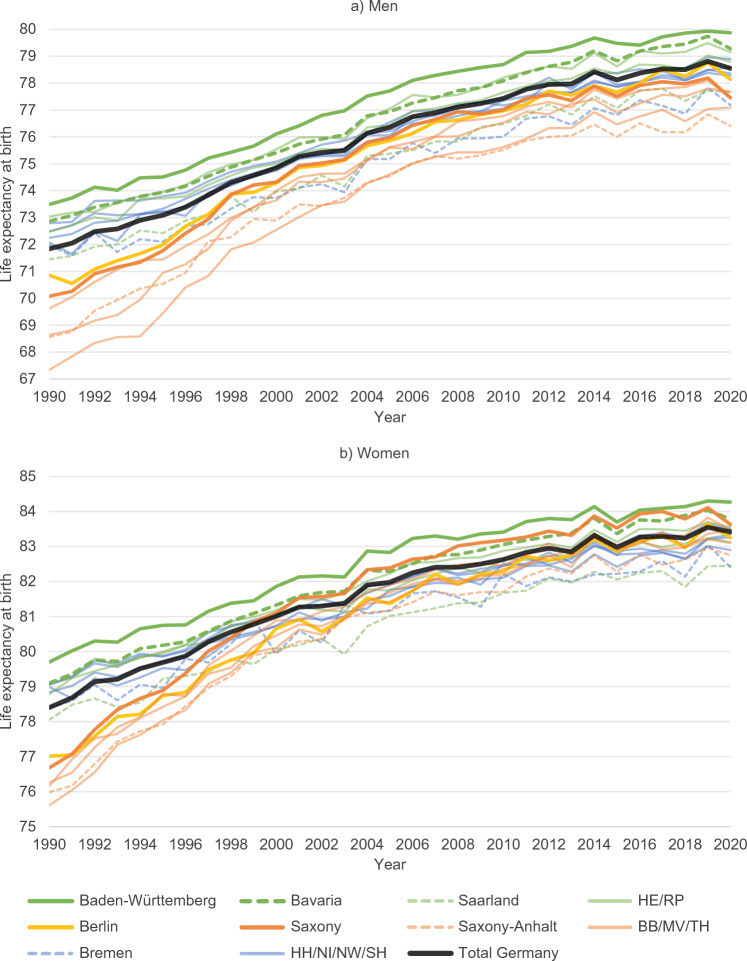
Life expectancy at birth in the German federal states by sex, 1990–2020. Abbreviations as for Fig. 2. Data source: German Federal and State Statistical Offices; authors’ calculations

Figure 1 shows the trend in life expectancy at birth in Germany’s federal states from 1990 to 2020, with the north-western states highlighted in blue, the states located in the south of western Germany in green, the capital of Berlin in gold and the eastern states in orange colours. For visibility, only the federal states with the most striking trends and patterns are illustrated in an easily distinguishable way. In general, the figure shows that there are considerable gradients between the north and the south and between the east and the west, with the latter being, however, only evident among men, not among women anymore. Baden-Württemberg and Bavaria in the south of Germany show particularly high levels of life expectancy, whereas Saxony-Anhalt, Bremen and Saarland show comparatively low levels. Life expectancy was increasing in all states before the onset of the COVID-19 pandemic, but at a slow pace, especially after 2014. There were serious decreases of life expectancy in 2015 and 2018, mainly caused by influenza epidemics. Most states even experienced higher losses in life expectancy from 2014 to 2015 than from 2019 to 2020. However, it must be considered that Germany experienced a relatively high increase in life expectancy before the decline in 2015. While most federal states experienced only moderate decreases in 2020, Saxony in the south of eastern Germany, however, showed a comparatively high loss in life expectancy (-0.76 years among men, -0.54 years among women).

**Fig. 2:**
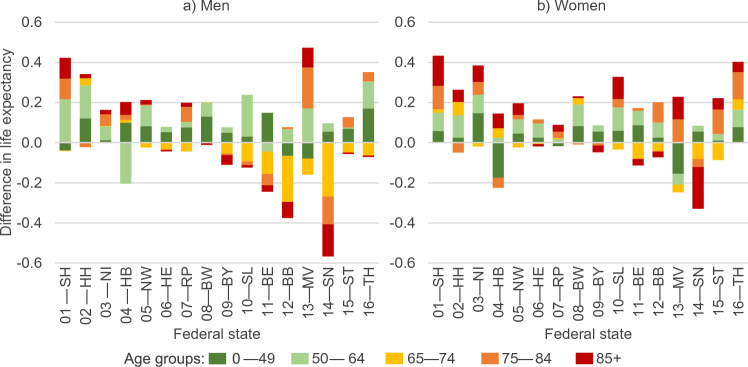
Life expectancy differences in the German federal states between 2020 and 2015/2019 decomposed into the contributions of selected age groups. Notes: Federal states are abbreviated as follows: 01: Schleswig–Holstein (SH), 02: Hamburg (HH), 03: Lower Saxony (NI), 04: Bremen (HB), 05: North Rhine-Westphalia (NW), 06: Hessen (HE), 07: Rhineland-Palatinate (RP), 08: Baden-Württemberg (BW), 09: Bavaria (BY), 10: Saarland (SL), 11: Berlin (BE), 12: Brandenburg (BB), 13: Mecklenburg-Vorpommern (MV), 14: Saxony (SN), 15: Saxony-Anhalt (ST), 16: Thuringia (TH). Data source: German Federal and State Statistical Offices; authors’ calculations

Figure 2 shows the differences in life expectancy between 2020 and 2015/2019 in the German federal states decomposed into the contributions of broad age groups. In general, decreases in life expectancy were more pronounced among men than among women. Among men, the older age groups from 65 upwards contributed most to excess mortality in those states affected by life expectancy losses. Among women, however, the same was only true for Saxony, revealing a more diverse age pattern, even though Berlin and Brandenburg experienced elevated mortality at ages 65–74 and 85 + and Bavaria at ages 75 + among women, too. Mortality at ages below 65 decreased in most states for both men and women. The smallest German federal state, consisting of the north-western German cities of Bremen and Bremerhaven, is an outlier to this pattern, showing excess mortality for men aged 50–64 and women aged 0–49.

### Spatial Distribution of Excess Mortality

**Fig. 3:**
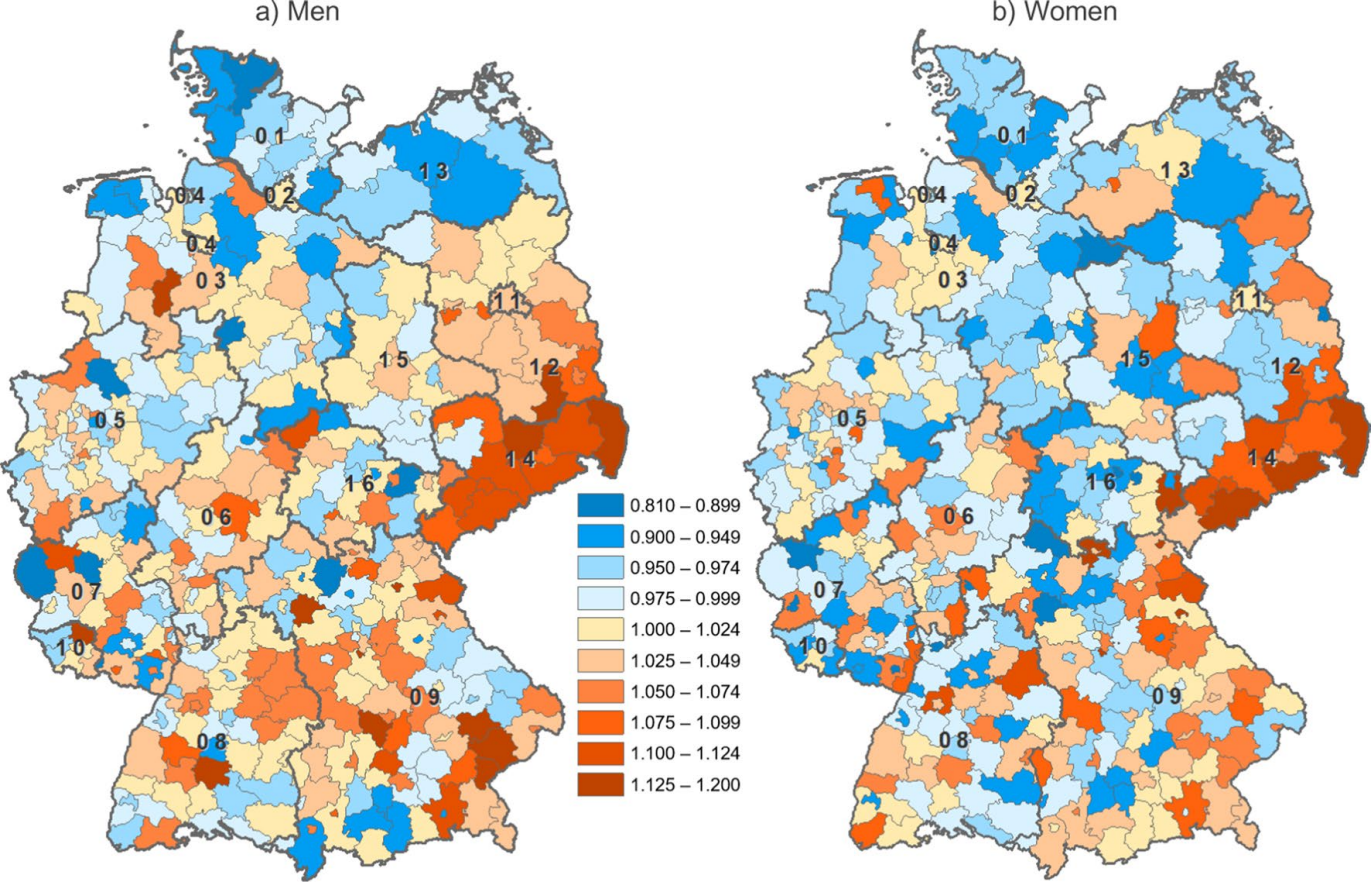
Standardised mortality ratios in Germany on the district level by sex in 2020 (with 2015/2019 as baseline). Notes: Abbreviations as for Fig. 2. Data source: German Federal and State Statistical Offices; authors’ calculations. Base map: © Federal Agency for Cartography and Geodesy (BKG)

Focusing on a smaller spatial level, Fig. 3 shows SMRs for Germany’s 400 districts by sex in 2020 as compared to the 2015/2019 baseline period, with bluish colours highlighting areas with reduced mortality levels (by up to 19%) and orange colours showing regions with increased mortality (by up to 20%). Among women, there were significantly more districts throughout Germany with decreased mortality than among men. Nevertheless, for both men and women, regions with high excess mortality were widely concentrated in Saxony (state number 14), south-eastern and north-eastern Bavaria (09) as well as south-eastern Brandenburg (12), whereas mortality decreased in wide parts of northern Germany. However, there are several outliers from this pattern, showing a remarkable extent of heterogeneity within Germany’s federal states. For instance, Bavaria and Baden-Württemberg (08) in southern Germany show districts on both ends of the range. There are also certain districts that rank at diametrically opposed ends of the range for men and women, respectively. For example, the districts of *Kitzingen* in Bavaria, *Nordhausen* in Thuringia (16) and *Vulkaneifel* in Rhineland-Palatinate (07) show excess mortality for men but reduced mortality for women. On the contrary, the districts of *Wittmund* in Lower Saxony (03), *Schwerin* in Mecklenburg-Vorpommern (13) and *Aschaffenburg-Land* in Bavaria show elevated mortality for women but decreased mortality for men. For more detail, Figure S1 (see online supplementary file 1) shows the SMRs with confidence intervals for the 30 most affected districts by sex.

### Cause-of-Death Patterns

**Fig. 4:**
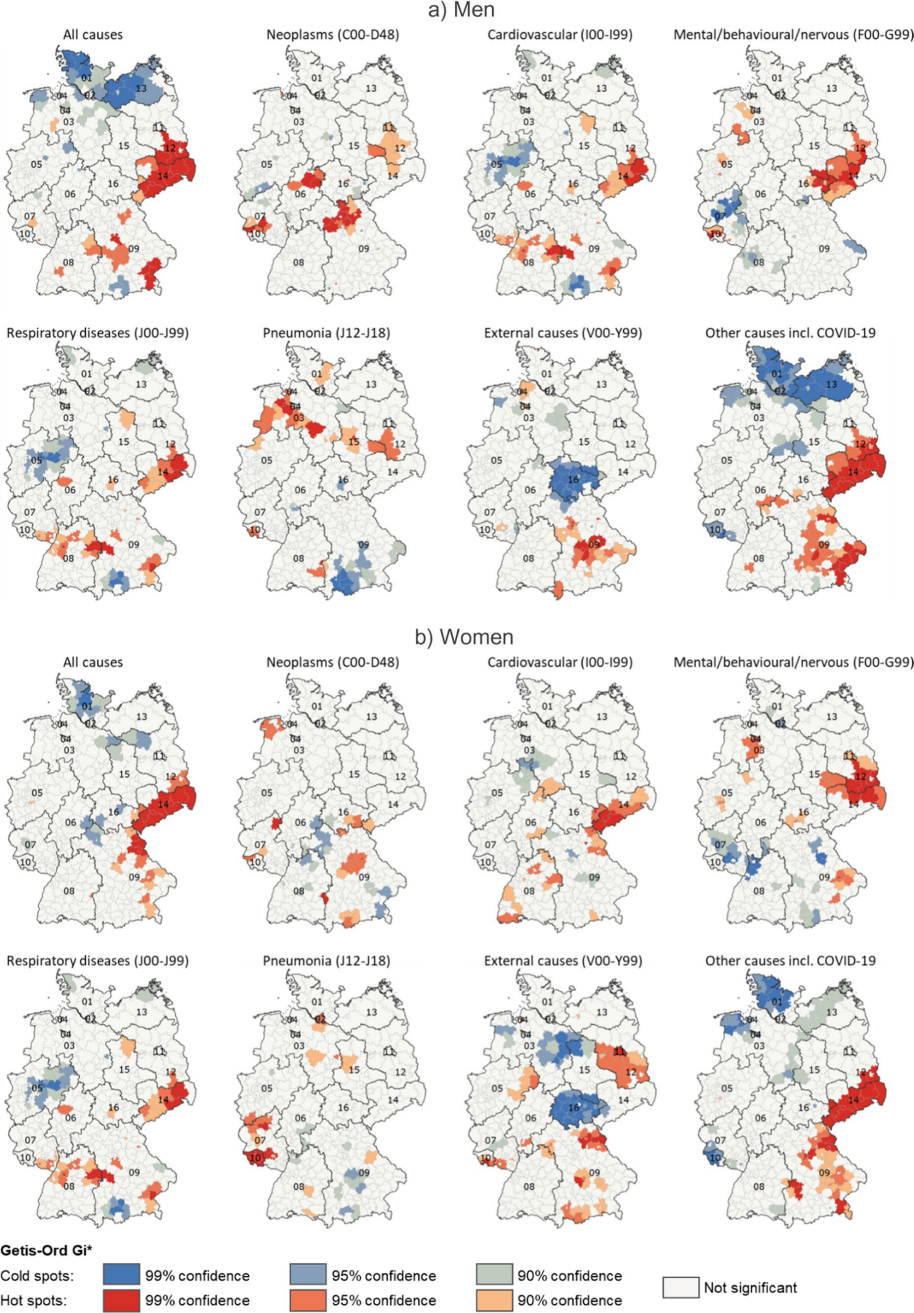
Hot and cold spots of excess mortality as identified by Getis-Ord Gi* statistic in 400 German districts by sex, selected causes, 2020. Notes: Abbreviations as for Fig. 1. Data source: German Federal and State Statistical Offices; authors’ calculations. Base map: © Federal Agency for Cartography and Geodesy (BKG)

From a cause-specific perspective, Fig. 4 shows the spatial hot and cold spots identified by Getis-Ord Gi* statistic based on the ratios of cause-specific SDRs from 2020 and 2015/2019. It reveals that the changes in all-cause mortality in the German districts were widely driven by the group of other diseases, which include COVID-19 in 2020. In addition, excess mortality in the most affected regions was also connected with increased rates in cardiovascular and mental/nervous diseases. Some of these regions showed elevated mortality from respiratory diseases as well. What is also striking is the reduction in external causes in Thuringia in central Germany. These patterns were true for both men and women.

**Fig. 5:**
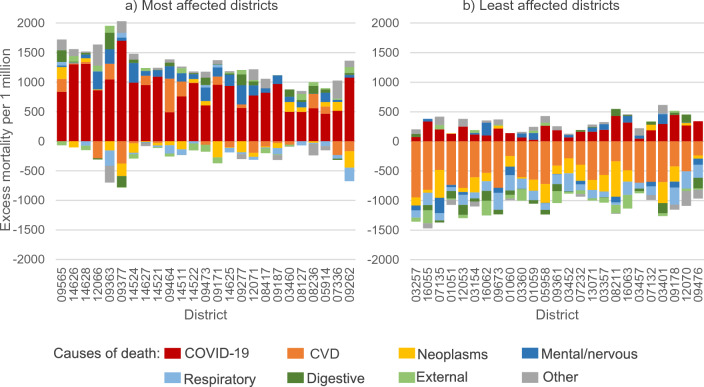
Difference in cause-specific standardised death rates between 2020 and 2015/2019 in the 25 most affected and 25 least affected German districts. Notes: The first two digits of the district number signal the federal state the district is located in (abbreviations as for Fig. 2). The official district classification is available at DESTATIS [30] and an administrative map at BKG [31]. Data source: German Federal and State Statistical Offices; authors’ calculations

Figure 5 shows the difference in cause-specific SDRs between 2020 and 2015/2019 in the 25 districts that showed the highest excess mortality and in the 25 districts that showed the highest mortality reduction. We combined both sexes because the regional patterns for men and women were widely similar, as also indicated by Fig. 4. In the most affected districts, the difference between 2020 and 2015/2019 was driven widely by the onset of COVID-19, especially in Saxon districts (identifiable by the state number of 14 as the first two digits), but also to a lesser extent by mental/nervous diseases. Many of these regions show elevated mortality from cardiovascular (CVD), digestive and other diseases as well. The least affected regions, however, experienced low COVID-19 mortality, less significant mortality from mental/nervous diseases and stronger decreases in cardiovascular diseases, neoplasms and respiratory diseases.

### Seasonal Patterns by Cause

Figure S2 (see online supplementary file 1) shows the distribution of cause-specific deaths by month and federal state. These seasonality indices reveal a winter peak, particularly in February and March, and a summer low from May to September. This is true for both periods, 2015/2019 and 2020, but not for all causes of death: For neoplasms and digestive diseases, this pattern is rather weak, especially in 2020, while external causes do not show a systematic seasonal pattern at all across regions. In 2020, the first wave of COVID-19 (from March to May) was particularly pronounced in Saarland, Bavaria and Baden-Württemberg, while eastern German states, such as Saxony, were hardly affected. The second wave (beginning in October) shows a diametrically opposed regional pattern, with Saxony showing a seasonality index of 9.3 in December, meaning the risk of dying from COVID-19 was about nine times (or by 830%) higher in December compared to the overall annual risk for Saxony. However, cardiovascular diseases – such as ischaemic heart (53%), cerebrovascular (40%) and other cardiovascular diseases (45%) – as well as mental/nervous diseases (60%) peaked in December in Saxony, too, showing significantly higher risks compared to previous months and previous years. Another striking region is Saarland, which shows extraordinarily high values for pneumonia (137%) and other respiratory diseases (77%) in March of 2020.

### Area-Level Associations

Based on supplementary spatial regressions, we found small but statistically significant positive correlations of all-cause excess mortality in 2020 with population density and proximity to the European hot spots of the second wave (see Table S2 in online supplementary file 2). Moreover, we found a negative correlation of all-cause excess mortality with hospital beds per capita, i.e. mortality decreased with an increasing number of available hospital beds on the district level. For COVID-19 (Table S1) and excess mortality from ‘other diseases’, we found the same association. Whereas socioeconomic area-level deprivation is the most important driving factor of spatial variation in all-cause mortality (Table S1), it shows no significant correlation with the change of mortality from 2015/2019 to 2020 (Table S2). With regard to mortality from COVID-19, we found positive associations with the number of nursing home places per capita and proximity to hot spots as well as a negative correlation with hospital beds per capita. The proximity to hot spots was also positively correlated with excess mortality from cardiovascular, mental/nervous and other diseases, albeit the size of the effect was rather small in every model. In excess morality from mental/nervous diseases, the share of people in need of care showed a highly positive association, while in respiratory diseases, the number of nursing home places was more important.

## Discussion

### Overall Regional Pattern

Based on official individual-level cause-of-death statistics, our results show that the German hot spots of excess mortality in 2020 were primarily located along the south-eastern border in proximity to Czechia, Poland and northern Italy, whereas the coastal regions in the north of Germany in proximity to Denmark were least affected. The north of Italy was among the earliest and most affected areas in Europe during the first wave of COVID-19, whereas Czechia and Poland were European hot spots of the second wave in late 2020 [4].

More detailed analyses of our data by season reveal that south-eastern Bavaria, which is located at the German-Austrian border and close to northern Italy, was most affected in the first wave, whereas Saxony and south-eastern Brandenburg, that are close to Poland and Czechia, were considerably more affected in the second wave than in the first one, thus confirming previous research on the timing of the pandemic [1, 4, 9, 25].

The German federal states that are closest to the European hot spots of the first and second waves of COVID-19—Bavaria, Brandenburg and Saxony—showed excess mortality almost entirely at ages 65 and older. This is particularly true for men. For Germany as a whole, 94% of all COVID-19 deaths in 2020 were recorded in this age group, as additional analyses of our data show. These results confirm the strong connection of COVID-19 mortality with age: While young and middle ages are hardly affected, fatality increases significantly at older ages [3, 7, 32, 33].

### Cause-Specific Variations

The cause-of-death distribution in the German districts that were most affected by excess mortality in 2020 was widely dominated by COVID-19, especially in December. However, we also found—to a lesser extent – elevated mortality from mental/nervous and cardiovascular diseases in the most affected areas in December of 2020. This could be related to side effects from SARS-CoV-2 and/or to the psychosocial stress caused by the corona crisis and the policy measures, such as lockdowns and social distancing, taken in reaction to it. Previous research has already shown that policy measures that foster social isolation affect mental health negatively [34–37]. Most of the highly affected areas are shaped by selective emigration, economic deprivation and a high percentage of older people, as regional indicators from the INKAR database [38] show, which – in combination with the spread of the disease and the introduction of measures of social distancing—are likely to foster loneliness and depression, especially around Christmas, when people usually celebrate with family members.

The least affected districts experienced comparatively low COVID-19 mortality. Moreover, mortality from mental/nervous diseases was less significant and decreases in cardiovascular diseases were much more pronounced, thus hinting at a lower level of perceived psychosocial stress in these areas, possibly resulting—aside from the greater distance to European hot-spot areas—from a more favourable population composition in terms of employment, income, social environment and age, and thus a better health-related resilience, as compared to the most affected areas. In the context of the pandemic, resilience has been discussed to be a key factor against loneliness among older people [39]. In fact, our spatial regression results show that a higher share of people in need of care was correlated with higher mortality from mental/nervous diseases, thus supporting this suggestion.

Furthermore, the proximity to hot spots of the second wave (Poland and Czechia) fostered the number of COVID-19 infections [40] and excess deaths, not only from COVID-19 but also from mental/nervous, cardiovascular and other diseases. Regarding COVID-19, the association with variables related to population structure was weak due to the use of an age-standardised measure as outcome variable. The crude number of COVID-19 deaths, however, was positively correlated with the share of older people [8]. A higher number of available hospital beds was associated with lower mortality from COVID-19 and with lower all-cause and ‘other’ excess mortality. In previous research, a negative correlation of German excess mortality with the number of hospital beds has been found as well [8], thus indicating a possible role of lacking healthcare infrastructure in regions that were particularly challenged by the pandemic.

### Peculiarities

Assuming an effect on mortality during lockdown periods, we had a closer look on seasonal cause-specific patterns. Germany’s first lockdown went into effect on 21 March 2020 and lasted until early May 2020, after which restrictions were gradually eased [41], albeit at a different pace across Germany’s federal states, as shown in our detailed overview of COVID-19-related policy measures taken in the German federal states from 2020 to 2023 (see online supplementary file 3, includes both English and German versions). Following a rise in infections, on 2 November 2020, a second lockdown was introduced, which was ‘light’ in the beginning but became stricter as of 16 December 2020. While we found significant increases in mental/nervous and cardiovascular diseases during the second lockdown, as mentioned before, we could not find a similar effect for the first lockdown. In external causes, for instance, we found varying regional trends for April 2020: While Bremen experienced a rise in mortality, Hamburg and Thuringia in particular experienced a decrease. The federal state of Thuringia is especially interesting in this context as it constituted a cold spot of mortality from external causes in 2020. As additional analyses reveal, Thuringia experienced slight decreases in mortality from a variety of accidents, including falls, traffic accidents and treatment complications, and most pronouncedly in February and April. Shaped by its low-mountain landscape with many potentially dangerous roads and trails, usually lots of transit traffic and a comparatively high density of older people, the reduced mobility possibly had a more protective effect in this regard than in other regions. The decrease in treatment complications might be associated with a reduced number of risky medical interventions during the lockdown, possibly also resulting from a decrease in severe accidents. However, this is only true for the month of April, as in May and December, when the state was partially locked down as well, Thuringia experienced its highest levels of mortality from external causes.

Another ‘outlier’ is the federal state of Bremen, showing excess mortality at comparatively young ages (0–49 among women, 50–64 among men). Additional cause-specific analyses reveal that this is widely related to increased mortality from external causes and mental/nervous diseases and to summer peaks related to respiratory and digestive diseases. On the other hand, as Germany’s smallest federal state with a population of 676,000 inhabitants (as of 31 December 2021), fluctuation could also partly explain this.

### Limitations

We cannot exclude regional differences in coding practices related to COVID-19. For example, Saarland in south-western Germany was a cold spot in 2020 for ‘other causes’ (incl. COVID-19) but a hot spot for pneumonia, which often occurs following a severe COVID-19 infection [14]. Considering that excess mortality from pneumonia in this region was widely limited to March, when there was still uncertainty how to code the underlying disease when a SARS-CoV-2 infection was (likely) involved—the international guidelines of the World Health Organization [42] were not finalised until 20 April 2020 [43]—and testing capacities still had to be expanded, we assume that COVID-19 was under-reported in Saarland in early 2020 as compared to the rest of Germany. On the contrary, excess mortality in a few Saxon and Bavarian districts was driven so overwhelmingly by COVID-19, with little change in other causal groups, that we cannot rule out that COVID-19 was possibly over-reported in certain regions where the health system was particularly challenged.

From a methodological viewpoint, our analyses are explorative in nature. This is mainly because German individual-level cause-of-death statistics cannot be linked to other data sources. Thus, we cannot calculate the contribution of individual health-related behaviour and area-level differences, such as the timing and severity of policy measures, which were overall not too different across the German federal states (see supplementary file 3). We applied spatial regressions to explore area-level correlations that, however, have to be interpreted with caution, as they do not imply any causal relationships and the number of suitable covariates is limited.

## Conclusions

To our knowledge, this is the most comprehensive and spatially-detailed analysis of German excess mortality in 2020, revealing a highly diverse regional pattern, with many federal states showing both hot spot and cold spot areas. We show that the German regional pattern of excess mortality in 2020 widely mirrors the European pattern, with the most affected regions being located in the southeast and the least affected regions being widely concentrated in the north. We found that excess mortality in the German hot spots of 2020 was particularly high in December and closely connected with older ages and COVID-19. However, we also found that the most affected areas experienced elevated mortality from mental/nervous and cardiovascular diseases, thus hinting at an increased level of psychosocial stress caused by the spread of the pandemic and/or the implementation of policy measures to combat it. Therefore, our results demonstrate the necessity that policy measures have to be carefully considered whether the benefits outweigh potential risks, taking account of varying demographic and socio-economic conditions across regions [44]. To do so in time, it is however essential to accelerate the collection of detailed mortality data, so that national and local policymakers can be provided earlier with relevant information in case of emerging epidemics or crises [45, 46].

### Supplementary Information

Below is the link to the electronic supplementary material.

Supplementary file1 (PDF 680 KB)

Supplementary file2 (PDF 576 KB)

Supplementary file3 (XLSX 356 KB)

## Data Availability

The German individual-level cause-of-death statistics from 2015 to 2020 are accessible upon application (fee required) at the Research Data Centre of the Federal Statistical Office and the Statistical Offices of the Federal States, DOI: 10.21242/23211.2015.00.00.1.1.0 to 10.21242/23211.2020.00.00.1.1.0. Population and death counts by age, sex and district can be requested at the statistical offices of the respective German federal states. We are not allowed to share the data due to the German data protection rules.

## Abbreviations

COVID-19: Coronavirus disease 2019
NUTS: Nomenclature of Territorial Units for Statistics
CVD: Cardiovascular diseases
ICD: International Classification of Diseases
SMR: Standardised mortality ratio
SDR: Standardised death rate
OLS: Ordinary least squares
AIC: Akaike information criterion
UTM: Universal Transverse Mercator
INKAR: Indicators and maps on spatial and urban monitoring
SARS-CoV-2: Severe acute respiratory syndrome coronavirus type 2
