# Supplementary material for: Evaluating Spatial, Cause-Specific and Seasonal Effects of Excess Mortality Associated with the COVID-19 Pandemic: The Case of Germany, 2020
Source: J Epidemiol Glob Health. 2023 Aug 4;13(4):664–75. doi: 10.1007/s44197-023-00141-0 (PMC10686941; doi:10.1007/s44197-023-00141-0)
Supplement: Supplementary file 1 — Supplementary file1 (PDF 680 KB) [file 44197_2023_141_MOESM1_ESM.pdf]

Evaluating Spatial, Cause-Specific and Seasonal Effects of Excess Mortality Associated with the COVID-19 Pandemic: The Case of Germany, 2020

Supplementary File 1

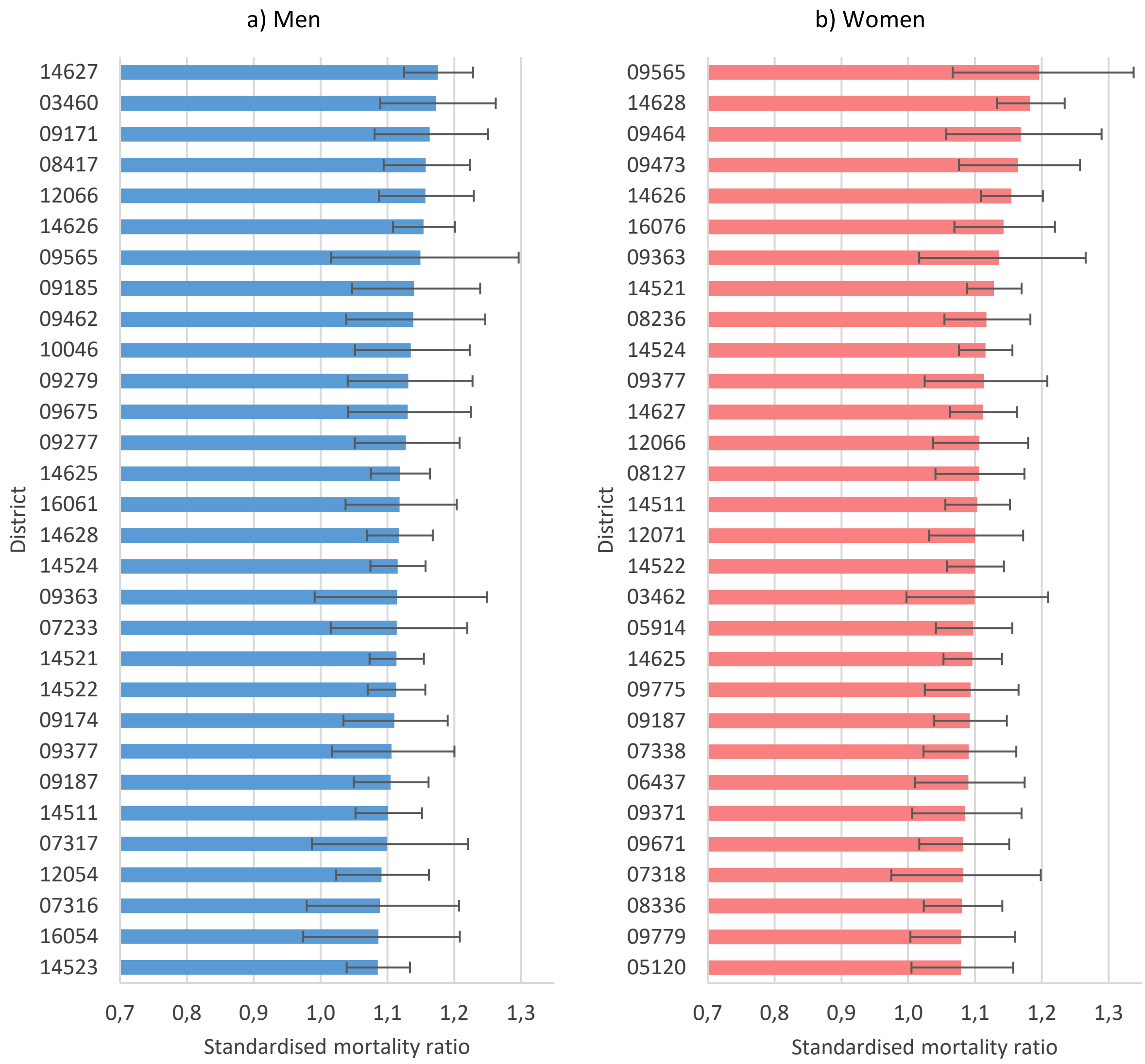

Fig. S1 Standardised mortality ratios by sex in 2020 (as compared to 2015/2019) in the 30 most affected German districts, with 95% confidence intervals. Notes: The first two digits of the district number signal the federal state the district is located in. 01: Schleswig-Holstein, 02: Hamburg, 03: Lower Saxony, 04: Bremen, 05: Northrhine-Westphalia, 06: Hessen, 07: Rhineland-Palatinate, 08: Baden-Württemberg, 09: Bavaria, 10: Saarland, 11: Berlin, 12: Brandenburg, 13: Mecklenburg-Vorpommern, 14: Saxony, 15: Saxony-Anhalt, 16: Thuringia. The official district classification is available at DESTATIS. A map of the German districts is available at BKG. Data source: German Federal and State Statistical Offices; authors' calculations.

Link to the full text article:
Mühlichen M, Sauerberg M, Grigoriev P. Evaluating Spatial, Cause-Specific and Seasonal Effects of Excess Mortality Associated with the COVID-19 Pandemic: The Case of Germany, 2020.
Journal of Epidemiology and Global Health 2023.
https://doi.org/10.1007/s44197-023-00141-0

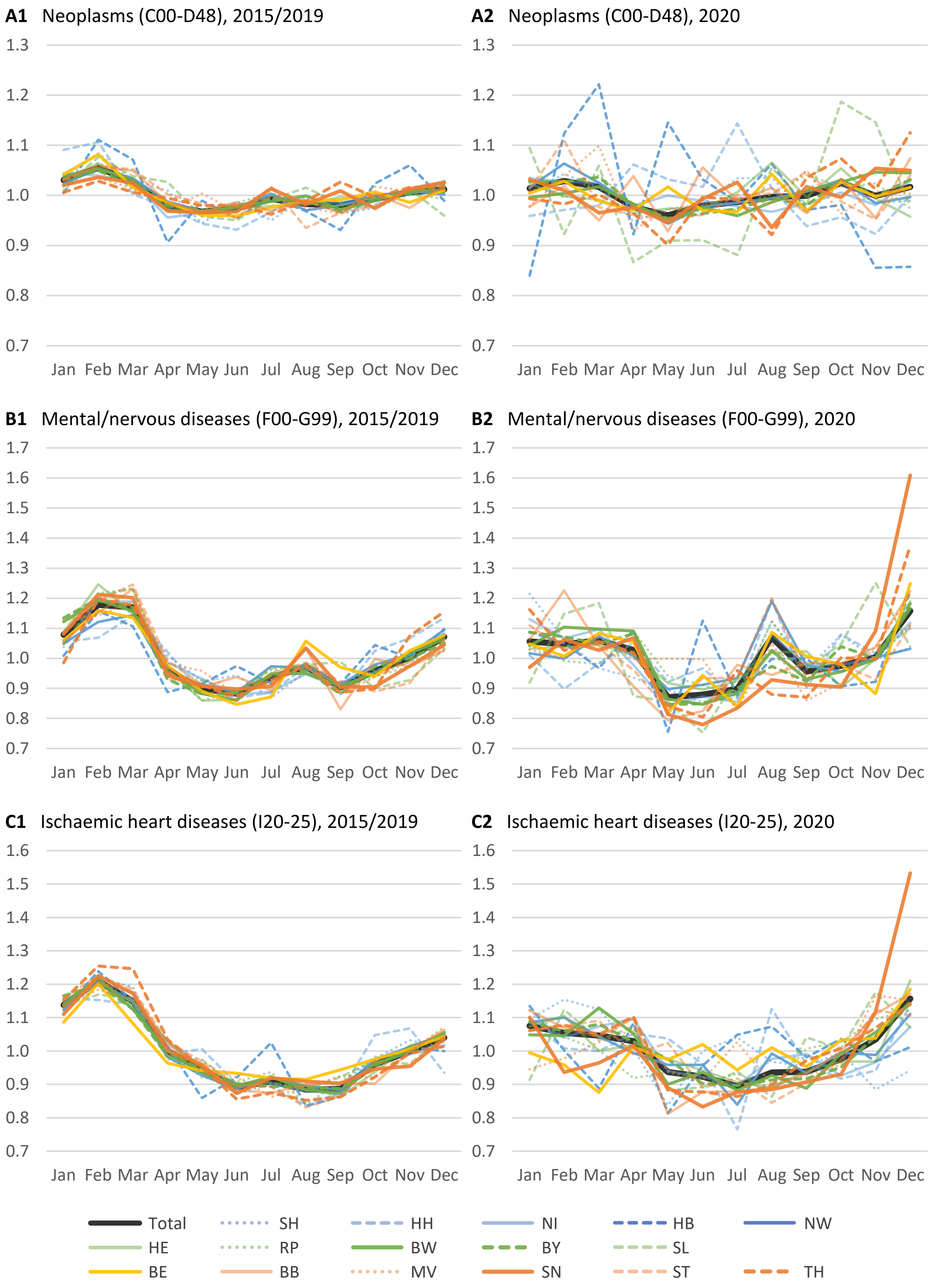

Fig. S2 Seasonality index of mortality by month and cause of death in the German federal states, 2015/2019 (left) and 2020 (right). Abbreviations: SH: Schleswig-Holstein, HH: Hamburg, NI: Lower Saxony, HB: Bremen, NW: Northrhine-Westphalia, HE: Hessen, RP: Rhineland-Palatinate, BW: Baden-Württemberg, BY: Bavaria, SL: Saarland, BE: Berlin, BB: Brandenburg, MV: Mecklenburg-Vorpommern, SN: Saxony, ST: Saxony-Anhalt, TH: Thuringia. Data source: German Federal and State Statistical Offices; authors' calculations. *(Part 1/4)*

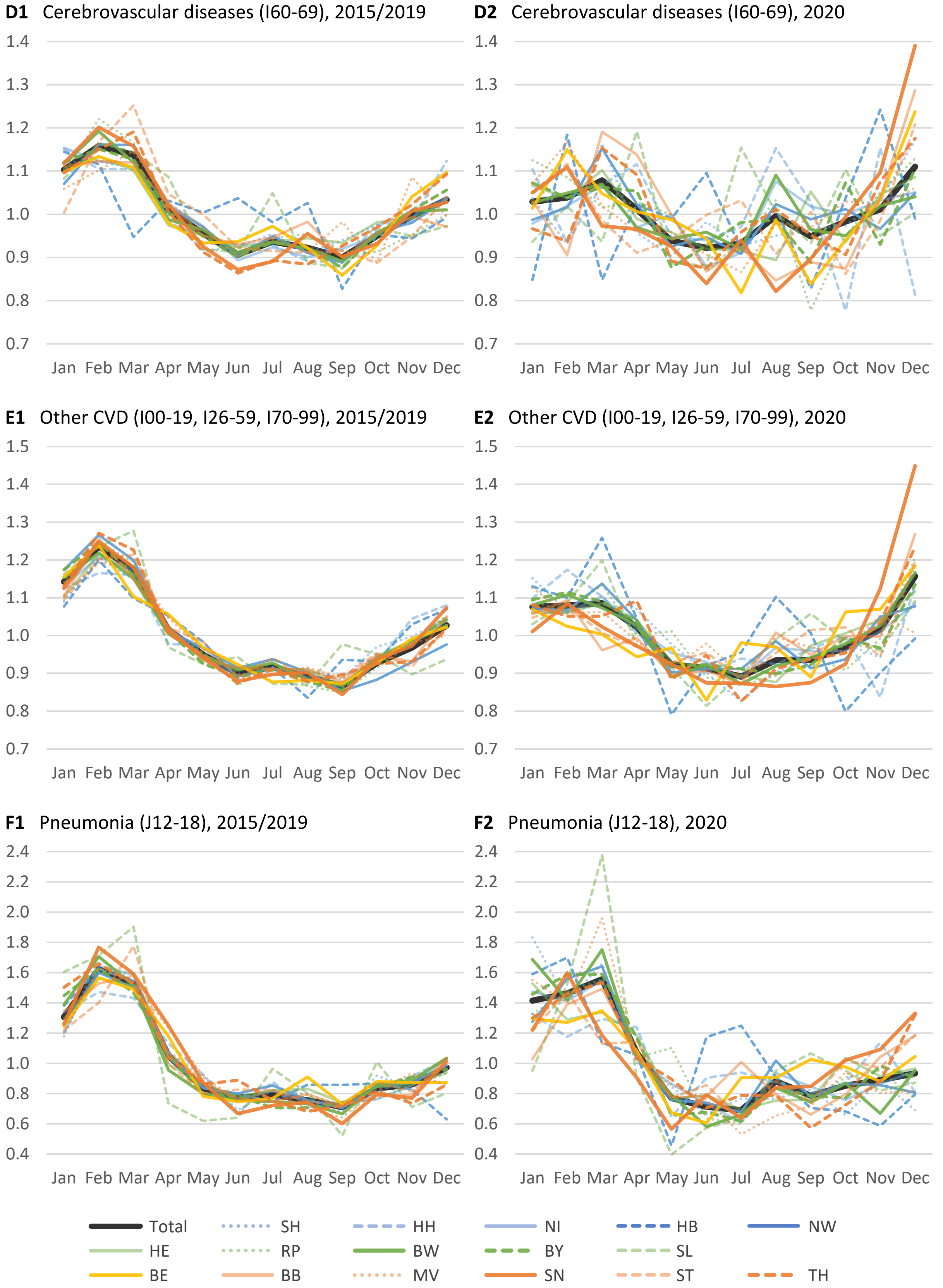

Fig. S2 Seasonality index of mortality by month and cause of death in the German federal states, 2015/2019 (left) and 2020 (right). Abbreviations: SH: Schleswig-Holstein, HH: Hamburg, NI: Lower Saxony, HB: Bremen, NW: Northrhine-Westphalia, HE: Hessen, RP: Rhineland-Palatinate, BW: Baden-Württemberg, BY: Bavaria, SL: Saarland, BE: Berlin, BB: Brandenburg, MV: Mecklenburg-Vorpommern, SN: Saxony, ST: Saxony-Anhalt, TH: Thuringia. Data source: German Federal and State Statistical Offices; authors' calculations. *(Part 2/4)*

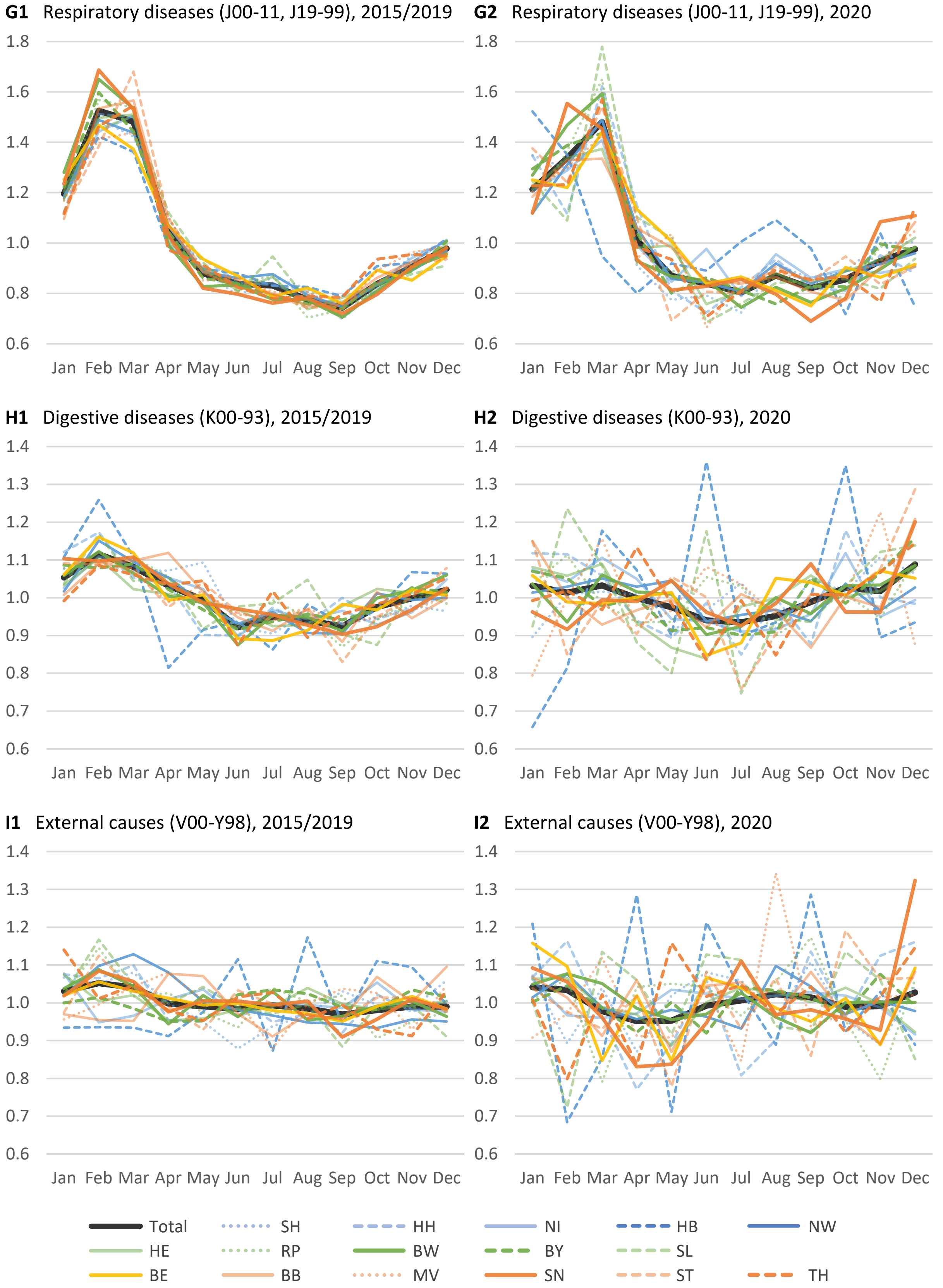

Fig. S2 Seasonality index of mortality by month and cause of death in the German federal states, 2015/2019 (left) and 2020 (right). Abbreviations: SH: Schleswig-Holstein, HH: Hamburg, NI: Lower Saxony, HB: Bremen, NW: Northrhine-Westphalia, HE: Hessen, RP: Rhineland-Palatinate, BW: Baden-Württemberg, BY: Bavaria, SL: Saarland, BE: Berlin, BB: Brandenburg, MV: Mecklenburg-Vorpommern, SN: Saxony, ST: Saxony-Anhalt, TH: Thuringia. Data source: German Federal and State Statistical Offices; authors' calculations. *(Part 3/4)*

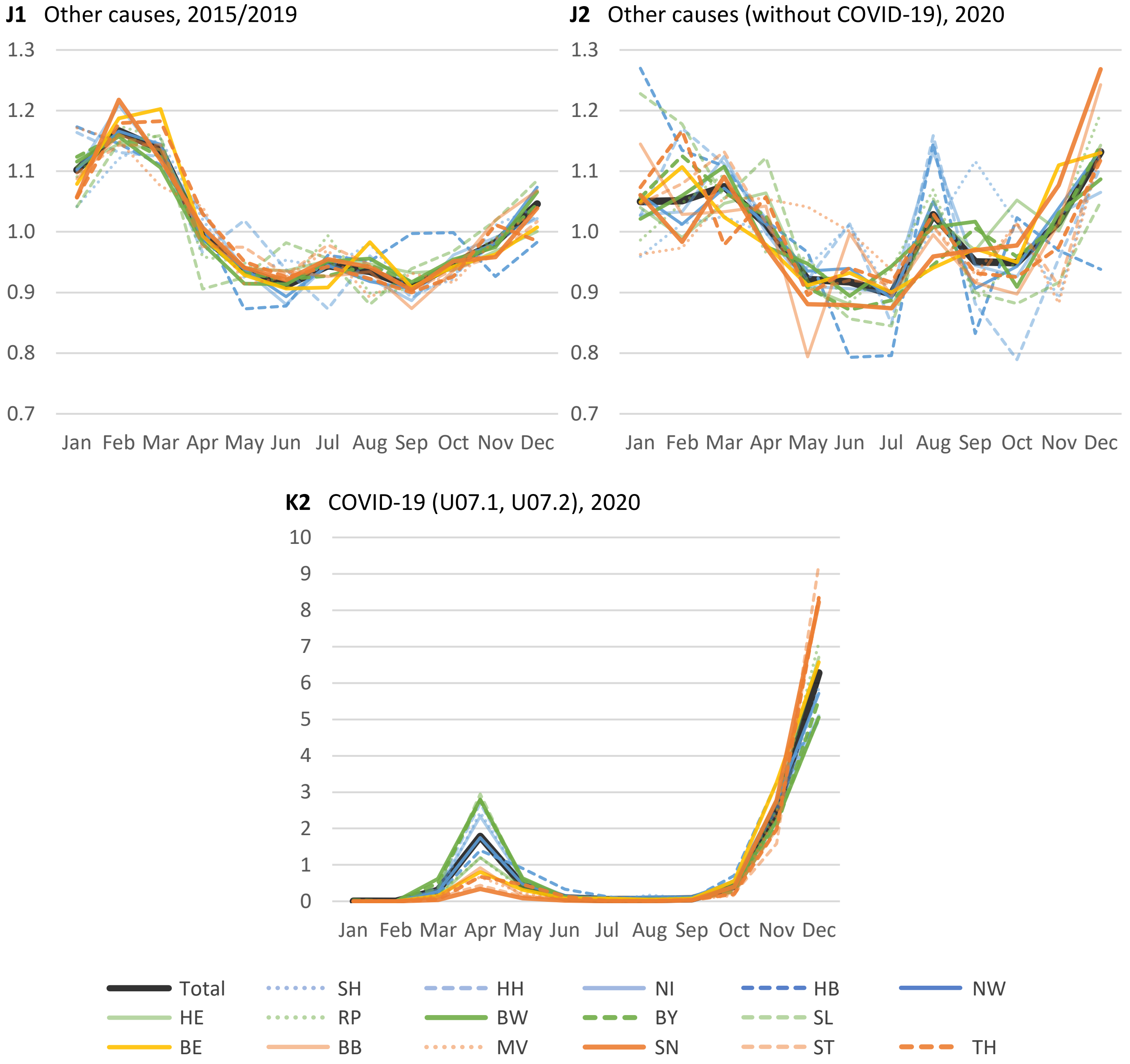

Fig. S2 Seasonality index of mortality by month and cause of death in the German federal states, 2015/2019 (left) and 2020 (right). Abbreviations: SH: Schleswig-Holstein, HH: Hamburg, NI: Lower Saxony, HB: Bremen, NW: Northrhine-Westphalia, HE: Hessen, RP: Rhineland-Palatinate, BW: Baden-Württemberg, BY: Bavaria, SL: Saarland, BE: Berlin, BB: Brandenburg, MV: Mecklenburg-Vorpommern, SN: Saxony, ST: Saxony-Anhalt, TH: Thuringia. Data source: German Federal and State Statistical Offices; authors' calculations. *(Part 4/4)*
