# Supplementary material for: Evaluating Spatial, Cause-Specific and Seasonal Effects of Excess Mortality Associated with the COVID-19 Pandemic: The Case of Germany, 2020
Source: J Epidemiol Glob Health. 2023 Aug 4;13(4):664–75. doi: 10.1007/s44197-023-00141-0 (PMC10686941; doi:10.1007/s44197-023-00141-0)
Supplement: Supplementary file 2 — Supplementary file2 (PDF 576 KB) [file 44197_2023_141_MOESM2_ESM.pdf]

Evaluating Spatial, Cause-Specific and Seasonal Effects of Excess Mortality Associated with the COVID-19 Pandemic: The Case of Germany, 2020

Supplementary File 2

Tab. S1 Spatial correlation of standardised death rates (SDRs) with contextual variables; German districts (N = 400) in 2020; both sexes combined; spatial regression models using queen weights

a) All-cause mortality in 2020 (SDR)

	OLS model		Spatial lag model		**Spatial error model**	
	Coefficient	SE	Coefficient	SE	Coefficient	SE
Socioeconomic deprivation[a]	2812.71***	250.92	2434.31***	240.26	2803.14***	248.66
Population density[b]	0.10**	0.05	0.07	0.05	0.06	0.05
Average age[b]	-46.60*	24.23	-60.94***	22.39	-35.82	25.55
Hospital beds per 1000 inhabitants[b]	-36.51***	8.96	-33.11***	8.28	-27.84***	7.77
Nursing home places per 10000 inhabitants[b]	6.19***	1.29	5.98***	1.20	5.99***	1.31
People in need of care per 100 inhabitants[b]	104.71***	40.15	60.02	37.72	118.95***	46.13
Proximity to hot spots[c]	1.84***	0.28	1.33***	0.27	1.53***	0.46
Intercept	10193.08***	1005.49	7421.38***	1006.59	9533.94***	1088.62
Wald statistic	–	–	52.59***	–	97.91***	–
AIC	6226.2	–	6182.7	–	6152.7	–

b) Mortality from COVID-19 in 2020 (SDR)

	OLS model		Spatial lag model		**Spatial error model**	
	Coefficient	SE	Coefficient	SE	Coefficient	SE
Socioeconomic deprivation[a]	-103.21	90.89	19.51	67.78	72.99	79.01
Population density[b]	0.05**	0.02	0.03*	0.01	0.03	0.02
Average age[b]	-11.03	8.78	-9.51	6.55	-4.33	8.23
Hospital beds per 1000 inhabitants[b]	-6.12*	3.25	-5.47**	2.42	-5.48**	2.36
Nursing home places per 10000 inhabitants[b]	-0.21	0.47	0.36	0.35	0.89**	0.41
People in need of care per 100 inhabitants[b]	8.62	14.54	5.93	10.84	-5.96	15.34
Proximity to hot spots[c]	0.86***	0.10	0.32***	0.08	0.84***	0.22
Intercept	1127.39***	364.21	553.30**	274.15	684.43*	357.50
Wald statistic	–	–	281.85***	–	314.31***	–
AIC	5413.8	–	5237.4	–	5236.1	–

Notes:

Abbreviations: SDR = standardised death rate per 1 million; OLS = Ordinary least squares; SE = standard error; *** p < 0.01; ** p < 0.05; * p < 0.1.

[a] German Index of Socioeconomic Deprivation (GISD) for 2019 taken from https://github.com/lekroll/GISD.

[b] Contextual variables for 31 December 2019 taken from INKAR database: https://www.inkar.de.

[c] Calculated by the nearest distance in kilometres between the centroid of each spatial unit and the Czech or Polish border; values negated, so the areas closest to the border show the highest values.

Tab. S2 Spatial correlation of SDR ratios with contextual variables by cause-of-death group; German districts (N = 400) in 2020; both sexes combined; spatial regression models using queen weights

a) All-cause excess mortality in 2020 (SDR ratio [d]*)*

	OLS model		Spatial lag model		Spatial error model	
	Coefficient	SE	Coefficient	SE	Coefficient	SE
Socioeconomic deprivation [a]	-2.69	18.03	1.91	16.79	5.64	18.51
Population density [b]	0.006*	0.004	0.005	0.003	0.005	0.004
Average age [b]	-0.90	1.74	-1.17	1.62	-0.95	1.87
Hospital beds per 1000 inhabitants [b]	-1.76***	0.64	-1.79***	0.60	-1.60***	0.60
Nursing home places per 10000 inhabitants [b]	0.02	0.09	0.06	0.09	0.07	0.10
People in need of care per 100 inhabitants [b]	2.87	2.89	2.67	2.69	3.39	3.30
Proximity to hot spots [c]	0.12***	0.02	0.08***	0.02	0.12***	0.03
Intercept	1058.24***	7.23	666.97***	92.95	1046.96***	79.07
Wald statistic	–	–	39.95***	–	41.40***	–
AIC	4120.1	–	4085.2	–	4085.6	–

b) Excess mortality from neoplasms in 2020 (SDR ratio [d]*)*

	OLS model		Spatial lag model		Spatial error model	
	Coefficient	SE	Coefficient	SE	Coefficient	SE
Socioeconomic deprivation [a]	31.02	24.60	31.08	24.38	31.02	24.36
Population density [b]	0.00	0.01	0.00	0.00	0.00	0.00
Average age [b]	3.41	2.38	3.41	2.35	3.41	2.35
Hospital beds per 1000 inhabitants [b]	-0.26	0.88	-0.25	0.87	-0.26	0.87
Nursing home places per 10000 inhabitants [b]	-0.13	0.13	-0.13	0.13	-0.13	0.13
People in need of care per 100 inhabitants [b]	-4.82	3.94	-4.83	3.90	-4.82	3.90
Proximity to hot spots [c]	0.01	0.03	0.01	0.03	0.01	0.03
Intercept	849.64***	98.58	854.04***	119.34	849.57***	97.66
Wald statistic	–	–	0.004	–	0.0005	–
AIC	4368.3	–	4370.3	–	4370.3	–

c) Excess mortality from cardiovascular diseases in 2020 (SDR ratio [d]*)*

	OLS model		Spatial lag model		Spatial error model	
	Coefficient	SE	Coefficient	SE	Coefficient	SE
Socioeconomic deprivation [a]	6.40	23.90	6.54	23.37	11.82	24.58
Population density [b]	0.00	0.00	0.00	0.00	0.00	0.01
Average age [b]	-0.83	2.31	-1.12	2.26	-1.25	2.43
Hospital beds per 1000 inhabitants [b]	-1.04	0.85	-1.21	0.83	-1.21	0.84
Nursing home places per 10000 inhabitants [b]	0.20*	0.12	0.20*	0.12	0.19	0.13
People in need of care per 100 inhabitants [b]	0.06	3.82	0.74	3.74	1.66	4.15
Proximity to hot spots [c]	0.06**	0.03	0.05**	0.03	0.07**	0.03
Intercept	954.08***	95.78	786.86***	114.20	965.29***	101.59
Wald statistic	–	–	7.56***	–	8.37***	–
AIC	4345.3	–	4340.1	–	4339.7	–

d) Excess mortality from respiratory diseases in 2020 (SDR ratio [d])

	OLS model		Spatial lag model		Spatial error model	
	Coefficient	SE	Coefficient	SE	Coefficient	SE
Socioeconomic deprivation [a]	-27.10	47.61	-24.24	46.10	-24.16	49.20
Population density [b]	-0.01	0.01	-0.01	0.01	-0.01	0.01
Average age [b]	4.42	4.60	3.53	4.45	3.55	4.90
Hospital beds per 1000 inhabitants [b]	-1.45	1.70	-1.09	1.65	-0.98	1.65
Nursing home places per 10000 inhabitants [b]	0.63**	0.25	0.56**	0.24	0.60**	0.26
People in need of care per 100 inhabitants [b]	1.42	7.62	0.18	7.38	1.50	8.44
Proximity to hot spots [c]	-0.08	0.05	-0.06	0.05	-0.07	0.07
Intercept	598.61***	190.79	434.04**	191.53	639.69***	205.21
Wald statistic	–	–	14.36***	–	14.42***	–
AIC	4896.6	–	4885.8	–	4886.1	–

e) Excess mortality from digestive diseases in 2020 (SDR ratio [d])

	OLS model		Spatial lag model		Spatial error model	
	Coefficient	SE	Coefficient	SE	Coefficient	SE
Socioeconomic deprivation [a]	7.27	61.40	6.17	60.73	6.58	61.74
Population density [b]	-0.01	0.01	-0.01	0.01	-0.01	0.01
Average age [b]	0.18	5.93	0.35	5.86	0.50	6.01
Hospital beds per 1000 inhabitants [b]	0.49	2.19	0.51	2.17	0.46	2.17
Nursing home places per 10000 inhabitants [b]	0.17	0.32	0.19	0.31	0.21	0.32
People in need of care per 100 inhabitants [b]	1.60	9.83	1.43	9.72	1.46	10.05
Proximity to hot spots [c]	0.02	0.07	0.01	0.07	0.01	0.07
Intercept	981.35***	246.05	912.95***	254.03	962.74***	249.75
Wald statistic	–	–	0.64	–	0.63	–
AIC	5100.1	–	5101.5	–	5101.5	–

f) Excess mortality from mental/behavioural/nervous diseases in 2020 (SDR ratio [d])

	OLS model		Spatial lag model		Spatial error model	
	Coefficient	SE	Coefficient	SE	Coefficient	SE
Socioeconomic deprivation [a]	50.35	53.26	39.64	52.20	34.57	54.41
Population density [b]	0.03**	0.01	0.02**	0.01	0.02**	0.01
Average age [b]	-1.00	5.14	-1.65	5.04	-1.44	5.35
Hospital beds per 1000 inhabitants [b]	-2.94	1.90	-2.78	1.86	-2.67	1.87
Nursing home places per 10000 inhabitants [b]	-0.45	0.27	-0.39	0.27	-0.44	0.28
People in need of care per 100 inhabitants [b]	26.88***	8.52	23.98***	8.44	26.83***	9.06
Proximity to hot spots [c]	0.16***	0.06	0.14**	0.06	0.16**	0.07
Intercept	1019.92***	213.44	884.61***	219.07	1045.54	223.09
Wald statistic	–	–	5.72**	–	4.60**	–
AIC	4986.3	–	4982.3	–	4983.7	–

g) Excess mortality from external diseases in 2020 (SDR ratio [d])

	OLS model		Spatial lag model		**Spatial error model**	
	Coefficient	SE	Coefficient	SE	Coefficient	SE
Socioeconomic deprivation [a]	31.63	83.49	29.17	76.54	-2.97	85.19
Population density [b]	-0.02	0.02	-0.02	0.02	-0.04*	0.02
Average age [b]	-12.00	8.06	-8.65	7.40	-12.92	8.66
Hospital beds per 1000 inhabitants [b]	2.36	2.98	3.31	2.73	4.16	2.73
Nursing home places per 10000 inhabitants [b]	-0.19	0.43	-0.13	0.39	-0.04	0.45
People in need of care per 100 inhabitants [b]	-24.29*	13.36	-16.77	12.30	-13.50	15.36
Proximity to hot spots [c]	0.17*	0.09	0.09	0.09	0.14	0.14
Intercept	1712.26***	334.58	1064.13***	318.90	1687.61***	366.23
Wald statistic	–	–	51.58***	–	52.06***	–
AIC	5346.0	–	5301.6	–	5303.3	–

h) Excess mortality from other diseases [e] in 2020 (SDR ratio [d])

	OLS model		Spatial lag model		**Spatial error model**	
	Coefficient	SE	Coefficient	SE	Coefficient	SE
Socioeconomic deprivation [a]	-119.81	93.41	-53.97	80.17	-22.33	91.21
Population density [b]	0.03*	0.02	0.02	0.02	0.01	0.02
Average age [b]	-1.33	9.02	-3.71	7.74	-4.29	9.39
Hospital beds per 1000 inhabitants [b]	-8.70***	3.34	-8.29***	2.86	-8.19***	2.84
Nursing home places per 10000 inhabitants [b]	-0.10	0.48	0.15	0.41	0.60	0.48
People in need of care per 100 inhabitants [b]	13.11	14.95	14.51	12.83	7.30	17.03
Proximity to hot spots [c]	0.90***	0.10	0.43***	0.10	0.88***	0.18
Intercept	1639.12***	374.33	843.57**	333.31	1668.42***	400.98
Wald statistic	–	–	105.22***	–	112.78***	–
AIC	5435.7	–	5349.5	–	5349.3	–

Notes:

Abbreviations: SDR = standardised death rate per 1 million; OLS = Ordinary least squares; SE = standard error; *** p < 0.01; ** p < 0.05; * p < 0.1.

[a] German Index of Socioeconomic Deprivation (GISD) for 2019 taken from https://github.com/lekroll/GISD.

[b] Contextual variables for 31 December 2019 taken from INKAR database: https://www.inkar.de.

[c] Calculated by the nearest distance in kilometres between the centroid of each spatial unit and the Czech or Polish border; values negated, so the areas closest to the border show the highest values.

[d] SDR 2020 divided by SDR 2015–2019, multiplied by 1,000.

[e] Excluding COVID-19, which is shown separately as SDR in Table S1. A ratio with previous years is not possible for COVID-19 because the disease did not occur before 2020.

Link to the full text article:
Mühlichen M, Sauerberg M, Grigoriev P. Evaluating Spatial, Cause-Specific and Seasonal Effects of Excess Mortality Associated with the COVID-19 Pandemic: The Case of Germany, 2020.
Journal of Epidemiology and Global Health 2023.
https://doi.org/10.1007/s44197-023-00141-0
